# The Prevalence and Pattern of Congenital Anomalies Among Neonates Admitted in a Tertiary Care Hospital in the Andaman and Nicobar Islands: A Retrospective Study

**DOI:** 10.7759/cureus.47853

**Published:** 2023-10-28

**Authors:** Tera Sravani, Pragathesh Palaniappan, Vinitha Shaju

**Affiliations:** 1 Pediatrics, Andaman and Nicobar Islands Institute of Medical Sciences (ANIIMS), Port Blair, IND

**Keywords:** andaman and nicobar islands, pattern, prevalence, neonates, congenital anomalies

## Abstract

Introduction

Congenital anomalies (CAs) constitute a significant determinant of neonatal mortality in India, and past studies have elucidated their diverse clinical presentations across different geographic regions. Given the limited availability of region-specific data in our locale, this study was initiated to elucidate the prevalence and phenotypic manifestations of congenital anomalies in live births within the distinctive setting of the Andaman and Nicobar Islands, India.

Methods

This retrospective observational study was conducted at the Department of Pediatrics of the Andaman and Nicobar Islands Institute of Medical Sciences (ANIIMS), located in Port Blair, during a defined one-year period extending from June 2022 to May 2023. All live births presenting with congenital anomalies within this stipulated timeframe were systematically enrolled in the study, and data was meticulously extracted from their respective medical records. The study data was methodically collated and organized utilizing the EpiData software (EpiData Association, Odense, Denmark), followed by a comprehensive descriptive analysis executed through the application of the PSPP software (GNU Project, Boston, MA).

Results

Among the 1661 neonates admitted during the study's defined period, a total of 61 neonates (3.7%) were identified as having congenital anomalies. Among these anomalies, cardiovascular anomalies were found to be the most prevalent, succeeded by gastrointestinal and central nervous system anomalies. Notably, major congenital anomalies were discerned in 20 out of 61 neonates, constituting approximately 33% of the neonatal population with anomalies. Neonates afflicted with congenital anomalies displayed a mortality rate of 11.5%.

Conclusion

The effects of congenital anomalies on individuals, families, and the society are far-reaching. Early identification and timely referral and intervention are the key factors in decreasing the mortality and morbidity associated with congenital anomalies.

## Introduction

Congenital anomalies (CAs) exert a substantial influence on child mortality, particularly in neonates. According to the WHO Birth Defects Surveillance Manual, CAs encompass a wide spectrum of structural or functional abnormalities present at birth, originating during prenatal development [1]. These anomalies are classified into major and minor categories, with major CAs yielding significant medical, social, or cosmetic ramifications necessitating clinical intervention while minor CAs exhibit limited health implications and aesthetic concerns [1]. Globally, CAs contribute to nearly 240000 neonatal deaths, with a substantial proportion occurring in low-middle-income countries [2]. In India, CA incidence ranges from 0.84% to 2.9%, and they rank as the fourth leading cause (8.6%) of neonatal mortality and the sixth leading cause of under-five mortality [3-7]. Congenital heart defects and neural tube defects prominently feature as severe CAs [2]. Although there are plenty of CA literatures available in India, there is a paucity of CA-related data in the Andaman and Nicobar Islands. In light of this substantial health burden, our study endeavors to ascertain the prevalence and typology of CAs in live births within the unique context of the Andaman and Nicobar Islands.

## Materials and methods

This was a facility-based retrospective observational study conducted in the Department of Pediatrics of the Andaman and Nicobar Islands Institute of Medical Sciences (ANIIMS), a renowned tertiary care hospital and academic institution situated in Port Blair, the Andaman and Nicobar Islands, India, over a period of one year spanning from June 2022 to May 2023. Our Institute is the only referral center in the whole of the Andaman and Nicobar Islands with an annual delivery rate of 2500-3000, most of which are high-risk pregnancies. As a part of routine newborn examination, all neonates admitted in our neonatology unit are screened for CAs, and further evaluation is performed wherever indicated. The clinical details of the patients are recorded in their medical records, which were retrieved retrospectively for study purposes.

Approval was obtained from the Institutional Scientific Research Committee of ANIIMS, Port Blair, with reference number ANIIMS/IEC/2022-23/51. Since ours was a retrospective study, informed consent was waived off. All collected data was kept confidential, and only the research team members had access to them. The subjects' personal identifiers were not recorded. The data collected was only used for research purposes and was not shared with anyone else.

In our study, we adopted a universal sampling technique; i.e., all live neonates admitted to our neonatology unit, during the study period, were enrolled in the study, and stillbirths were excluded from the study. Medical records of all the live neonates admitted to our neonatology unit during the study period were reviewed, and those with structural CAs were included in this study. The data regarding the mother's age, antenatal risk factors, gestational age, birth weight, gender, mode of delivery, pattern of CAs, and outcome was extracted from the medical records and entered in a standardized proforma. Study data management was facilitated using the EpiData software (EpiData Association, Odense, Denmark), with subsequent analytical procedures carried out employing the PSPP software (GNU Project, Boston, MA) to generate informative descriptive statistics. The prevalence of CAs was estimated as the percentage of newborns with CAs to the total number of newborns admitted during the study period. Each study variable has been represented as the total number and percentage.

## Results

During the study period, 1661 neonates were admitted to our neonatology unit, among which 1168 were term and 493 were preterm. Out of 1661 neonates, 3.7% (n=61) had congenital anomalies (CAs). Of the study cohort, 57.4% were males, and 42.6% were females. Notably, CAs were more prevalent among neonates born to multigravida mothers (52.5%, n=32) and mothers aged 20-35 years (82%, n=50), with 16.4% (n=10) of study subjects born to mothers with gestational diabetes mellitus. In terms of birth characteristics, CAs were most frequently observed in term neonates (65.6%, n=40) and those with birth weights between 2.5 kg and 3.49 kg (52.5%, n=32). Predominantly, congenital heart defects (44%, n=27) and anomalies of the gastrointestinal system (16.4%, n=10) were the most prevalent types of CAs in our study. Among the 61 neonates with CAs, 33% (n=20) exhibited major CAs, and 23% (n=14) had multiple anomalies. Alarmingly, our study revealed a notable mortality rate of 11.5% (n=7/61) among neonates with CAs, which significantly impacts our overall neonatal mortality rates. The baseline characteristics, clinical profile of CAs of the study subjects, and pattern of CAs in term and preterm neonates are shown in Tables 1-3.

**Table 1:** Baseline characteristics of the study subjects LSCS: lower segment cesarean section

Variables	Frequency (n)	Percentage (%)
Gender		
Male	35	57.4
Female	26	42.6
Age of the mother		
<20 years	02	3.3
20-35 years	50	82.0
>35 years	9	14.7
Parity of mother		
Primigravida	29	47.5
Multigravida	32	52.5
Mode of delivery		
Normal delivery	23	37.7
LSCS	37	60.7
Assisted delivery	01	1.6
Antenatal risk factors		
Gestational diabetes mellitus	10	16.4
Pregnancy-induced hypertension	7	11.5
Gestational age		
<32 weeks	05	8.2
32-37 weeks	16	26.2
>37 weeks	40	65.6
Birth weight		
1-1.49 kg	06	9.8
1.5-2.49 kg	18	29.5
2.5-3.49 kg	32	52.5
>3.5 kg	05	8.2

**Table 2:** Profile of congenital anomalies in neonates PUJ: ureteropelvic junction

Congenital anomalies (CAs) (profile and outcome)	Frequency (n)
Cardiovascular system (n=27, 44.3%)	
Acyanotic	25
Cyanotic	02
Gastrointestinal system (n=10, 16.4%)	
Cleft palate	01
Anorectal malformation	04
Tracheoesophageal fistula	01
Hirschsprung disease	01
Small intestine atresia	03
Central nervous system (n=8, 13%)	
Meningomyelocele	02
Arnold-Chiari malformation	01
Congenital hydrocephalus	05
Genitourinary system (n=7, 11.5%)	
PUJ obstruction	04
Others	03
Musculoskeletal system (n=4, 6.5%)	
Congenital talipes equinovarus (CTEV)	02
Polydactyly	02
Respiratory system (n=1, 1.6%)	
Congenital lobar emphysema	01
Syndromes (n=9, 14.8%)	
Down syndrome	05
Others	04
Major CAs (n=20, 33%)	
Central nervous system	08
Cardiovascular system	02
Gastrointestinal system	09
Respiratory system	01
Outcome	
Discharged	52
Death	07
Leaving against medical advice (LAMA)	02

**Table 3:** Pattern of CAs in term versus preterm neonates CAs: congenital anomalies

CAs (system involved)	Term (n=40, 66%)	Preterm (n=21, 34%)
Central nervous system	3 (7.5%)	5 (24%)
Cardiovascular system	16 (40%)	11 (52%)
Gastrointestinal system	8 (20%)	2 (10%)
Genitourinary system	6 (14.5%)	1 (5%)
Musculoskeletal system	4 (6.5%)	0
Respiratory system	1 (2.4%)	0
Syndromic	2 (5%)	7 (35%)

## Discussion

In our study, we found the prevalence of CAs to be 3.7%. This is consistent with previous studies conducted in India by Seba et al. [3], Ghosh et al. [8], and Tiwari and Gupta [9] where they reported incidences of 2.9%, 2.14%, and 2.56% respectively. A meta-analysis conducted by Bhide and Kar showed a pooled prevalence rate of 184.48 CAs per 10000 live births [7]. Studies from North India and North Kerala also reported lower incidences of 1.7% and 0.84%, respectively [4,10]. In a retrospective study conducted by Kumar et al. over a period of 20 years (1998-2017), the prevalence rate was reported as 182 per 10000 live births [5]. Similarly, a study conducted in South India by Cherian et al. showed a prevalence of 12.5 per 1000 live births [11]. Studies have shown that CAs are most common in males [3,4,6], and our study also observed a higher prevalence of CAs in male subjects. The majority of our study subjects were born to multiparous mothers, which aligns with the findings reported by Seba et al. [3], Sarkar et al. [6], and Bhalerao and Bhalerao [12]. Furthermore, research by Seba et al. [3] and Ghosh et al. [8] indicated that CAs are commonly observed in mothers over the age of 30, while our study showed that most mothers of neonates with congenital anomalies were between 20 and 35 years of age. Similar findings were reported by Tiwari and Gupta [9]. Additionally, most of our study subjects were of term gestation (65.6%) and with birth weight ranging from 2.5 to 3.49 kg (52.5%). These findings were comparable with those reported by Sinha et al. [10].

Studies conducted by Seba et al. [3], Cherian et al. [11], and Bhalerao and Bhalerao [12] revealed that anomalies of the musculoskeletal system are the most prevalent form of CAs. A meta-analysis by Bhide and Kar showed similar results with anomalies of the musculoskeletal system being a common form of CAs in live births; however, when stillbirths were included, CAs of the central nervous system appeared as the most prevalent [7]. A study done by Prashar et al. showed that CAs are most commonly seen in the central nervous system, followed by the urinary system [13], whereas Kumar et al. [5] and Doddabasappa et al. [14] reported that CAs tend to occur more frequently in the circulatory system. As per the WHO's fact sheet on congenital disorders, congenital heart defects are the most common form of severe CAs [2]. In our study as well, the overall highest prevalence of CAs was observed in the cardiovascular system (44%), followed by the gastrointestinal system (16.4%) and the central nervous system (13%). These findings were comparable with the study conducted by Kumar et al. [5]. Among term neonates, CAs were commonly observed in the cardiovascular system, followed by the gastrointestinal system, whereas in preterm, the CAs were commonly seen in the cardiovascular system, followed by the central nervous system.

Among subjects with congenital heart diseases, 92% (n=25) had acyanotic heart disease, and 8% (n=2) had cyanotic heart disease. Our findings were consistent with other similar studies [6,8]. The most common congenital heart defect identified in our study was atrial septal defect. Among 27 newborns with congenital heart disease, two (7.4%) were born to mother aged less than 20 years, eight (30%) were born to mothers 20-25 years of age, seven (26%) were born to mothers 25-30 years of age, four (14.8%) were born to mothers 30-35 years of age, and six (22.2%) were born to mothers 35-40 years of age. The prevalence of congenital heart disease was high in neonates born to mothers 35-40 years of age (6/7, 86%), followed by mothers less than 25 years of age (10/21, 48%), compared to neonates born to mothers 25-35 years of age (11/33, 33%). The findings of our study have been corroborated by another study done by Mamasoula et al. in which they observed the combined prevalence of congenital heart diseases to be higher among newborns born to mothers 35-45 years of age and mothers less than 25 years of age [15].

In our study, congenital hydrocephalus was the most common form of CAs related to the central nervous system, followed by meningomyelocele. Among neonates with CAs related to gastrointestinal tract (GIT) (n=10), anorectal malformation (40%) was the most prevalent form, followed by atresia of the small intestine. We observed that nine (14.8%) of our subjects had syndromic conditions with Down syndrome being the most common (55%), and our findings are comparable with similar studies as well [8]. In our study, 33% of subjects had major CAs, whereas a study done by Pattanaik et al. showed a prevalence of 10.4% [16]. Major CAs were most commonly observed in the gastrointestinal system (45%), followed by the central nervous system (40%). In our study, 23% had multiple CAs. The average length of hospital stays for the subjects in our study ranged from seven to 14 days. Kumar et al. [5] and Vikram and Pushpa [17] reported CA-related death rate of 0.67% and 0.38%, while Tiwari and Gupta reported mortality of 30% in neonates with congenital birth defects [9]. In our study, the mortality rate among newborns with CAs was 11.5% (7/61) compared to 1.8% (29/1600) in newborns without any CAs. The overall neonatal mortality during the study period was 2.2% (36/1661), out of which one-fifth (19.4%, 7/36) was attributed to CAs. As a retrospective study conducted in a tertiary care setting, ours may not be a representative sample of the general population, and stillbirths were not included in the study. Moreover, few details such as folic acid consumption and consanguinity are missing in most of the records, because of which the correlation of these factors with CAs could not be analyzed. These were the few limitations of our study. The Ministry of Health and Family Welfare of the Government of India has recognized the importance of birth defects and has taken proactive measures through the Rashtriya Bal Swasthya Karyakram (RBSK) program. As part of this program, comprehensive newborn screening for birth defects has been implemented at all delivery points, with a particular focus on visible birth defects [18]. When these conditions are identified, affected infants are referred to specialized facilities for further evaluation and treatment.

## Conclusions

The profound impact of congenital anomalies (CAs) reverberates through the lives of affected individuals, their families, and the society at large. The pressing imperative of our time is to embark on comprehensive, large-scale, prospective research to discern the multifaceted factors contributing to CAs and to unveil the logistical elements shaping their outcomes. Mitigating this burden necessitates a holistic approach, encompassing public awareness campaigns, the identification of treatable etiologies, the provision of adequate antenatal care, robust prenatal counseling, the early detection of CAs, expedited referral systems, and timely interventions. The effective implementation of programs such as the Rashtriya Bal Swasthya Karyakram (RBSK) can collectively drive a reduction in the disease burden associated with CAs within our communities. Based on the findings of our retrospective study on the prevalence and patterns of congenital anomalies among neonates in the Andaman and Nicobar Islands, several key recommendations emerge. Firstly, there is a pressing need for the establishment of region-specific prenatal screening programs to facilitate the early detection of congenital anomalies. This would enable timely interventions, enhancing neonatal outcomes. Secondly, efforts should be directed toward strengthening neonatal care facilities in the region to effectively manage major congenital anomalies, placing an emphasis on specialized care and surgical interventions when necessary. Lastly, a collaborative approach with healthcare authorities is essential to implement public awareness campaigns that emphasize the importance of antenatal care, prenatal counseling, and the early diagnosis of congenital anomalies. This will ensure that expectant mothers are well-informed and receive the necessary support to reduce the burden of congenital anomalies in the islands.
